# Supplementary material for: Multichannel Compressive Sensing MRI Using Noiselet Encoding
Source: PLoS One. 2015 May 12;10(5):e0126386. doi: 10.1371/journal.pone.0126386 (PMC4429034; doi:10.1371/journal.pone.0126386)
Supplement: S1 Matlab Code — (ZIP) [file pone.0126386.s001.zip › code/irt/data/pet,trans,2d,sino/index.html]

Fessler, Jeffrey A.: PET Data


# PET Data: Try your favorite algorithm!

This page provides free access to some PET sinogram data.
You can use this data in any way you wish, no strings attached.
If you publish results using this data
(on paper, electronically, or whatever),
it would be appropriate to acknowledge its source.

---


## PET Thorax Phantom, Transmission Scan, CTI ECAT EXACT System

  
The left image is the "blank" scan,
and the right image is the transmission scan.
  
For FBP reconstruction,
take the ratio of transmission over the blank scan,
smooth the resulting sinogram radially,
take the negative logarithm,
apply the ramp filter,
and then backproject.
Here is what you should get:


All this is easily done using the
 ASPIRE software package.

For better results,
I recommend the
grouped-coordinate ascent algorithm
maximizing a penalized-likelihood objective function,
also available in ASPIRE.
Here is what you get:


To access the raw data,
 click here for the blank data
and
 click here for the transmission data.
  
Probably you want to shift-click.

Blank data:

- 4 byte IEEE floating point values in SUN byte order- 160 radial samples (varies fastest) by 192 angular samples (over 180 degrees)- range of values: 30.76 to 98.28, sum is 1.75756e+06

Transmission data:

- 2 byte signed integers in SUN byte order.- 160 radial samples (varies fastest) by 192 angular samples (over 180 degree- range of values: 0 to 130, sum is 920653

The attenuation coefficient of water at 511 keV is about 0.1 / cm,
and the phantom is mostly water except for the teflon spine
and the styrofoam lungs.

---

This data came from an Siemens/CTI ECAT/EXACT PET scanner.
Details about this scanner are described in:
"Performance evaluation of a new generation positron scanner ECAT EXACT,"
K Wienhard L Eriksson S Grootoonk M Casey U Pietrzyk W D Heiss,
JCAT, 16(5):804-13, Sep. 1992.
(J. Comput. Aid. Tomo.)

---

 Back to Fessler home page
