# Supplementary material for: Multichannel Compressive Sensing MRI Using Noiselet Encoding
Source: PLoS One. 2015 May 12;10(5):e0126386. doi: 10.1371/journal.pone.0126386 (PMC4429034; doi:10.1371/journal.pone.0126386)
Supplement: S1 Matlab Code — (ZIP) [file pone.0126386.s001.zip › code/irt/doc/doc.pdf]

Image Reconstruction Toolbox for MATLAB (and Freemat)

Jeffrey A. Fessler
University of Michigan
fessler@umich.edu

September 16, 2011

1 Introduction

This is an initial attempt at documentation of the image reconstruction toolbox (IRT) for MATLAB, and any other MATLAB emulator that is sufficiently complete. This documentation is, and will always be, hopelessly incomplete. The number of options and features in this toolbox is ever growing.

2 Overview

Although there are numerous options in the IRT, most image reconstruction examples in the toolbox have the following outline. A concrete example is `example/recon_limited_angle1.m`.

- Pick an image size and generate a "true" digital phantom image such as the Shepp-Logan phantom.
- Generate a system matrix (usually called `A`), typically a `fatrix2` object (see below), that will be used for iterative reconstruction.
- Generate simulated measurements `y`, possibly using the `fatrix2` object or possibly using an analytical model (*e.g.*, the line-integrals through the phantom). (Using the `fatrix2` object is cheating because in the real world there is model mismatch that contaminates the measurements.)
- Perform a conventional non-iterative reconstruction method (*e.g.*, `fbp2`) to get a baseline image for comparison.
- Generate a regularization object (usually `R`).
- Check the predicted resolution properties of that `R` using `qpwls_psf`, and adjust the regularization parameter β if necessary.
- Apply an iterative algorithm to the data `y` using the system model `A` and the regularizer `R` for some user-specified number of iterations.

2.1 Getting started

The best way to learn is probably to run an example file like `recon_limited_angle1.m`, possibly inserting `keyboard` commands within the m-file to examine the variables. Most of IRT routines have a built-in help message; *e.g.*, typing `im` without any arguments will return usage information. Many IRT routines have a built-in test routine, *e.g.*, `ellipse_sino test` runs a built-in test of the routine for generating the sinogram of ellipse objects. The test code illustrates how to use the routine.

2.2 Masking

One subtle point is that we usually display images as a rectangular grid of `nx` × `ny` pixels, but usually the iterative algorithms work on column vectors. Often only a subset of the pixels are updated, as illustrated in Fig. 1. A logical array called the `mask` specifies which pixels are to be updated. The function call `x_col = x_array(mask(:))` extracts the relevant pixel elements "within the mask" into a column vector. Conversely, the call `x_array = embed(x_col, mask)` puts the elements of the column vector back into the appropriate places in the array.

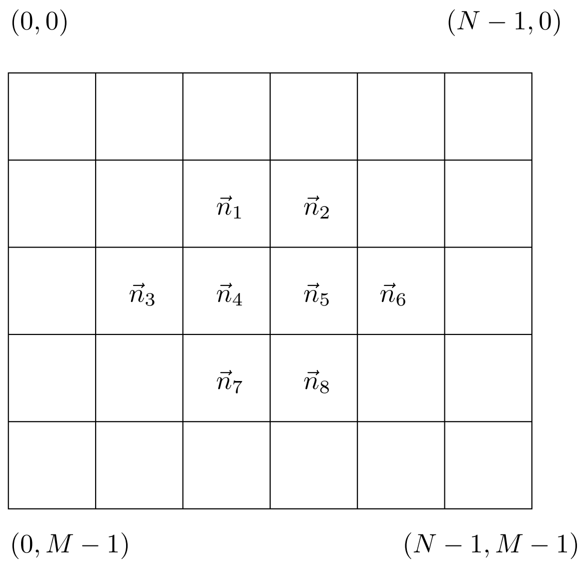

Figure 1: A $N \times M = 6 \times 5$ lattice with approximately elliptical FOV. Only the $n_{\mathrm{p}} = 8$ pixels with indices are estimated. In this example, $n_{\mathrm{p}} = \sum_{n=0}^{N-1} \sum_{m=0}^{M-1} \chi[n, m] = 8$.

The following code illustrates these ideas.

```
mask = false(6,5); mask([9 10 14:17 21 22]) = true;
xc = [10:5:45]';
xa = embed(xc, mask)
```

Here is the resulting output.

```
0     0     0     0     0
0     0    20     0     0
0    10    25    40     0
0    15    30    45     0
0     0    35     0     0
0     0     0     0     0
```

Conversely, typing the following produces a vector that is the same as the original `xc` vector.

```
xa(mask)
```

3 Special structures

The IRT uses some special custom-made object classes extensively: the `strum` class, which provides structures with methods, and the `fatrix2` class (and its obsolete predecessor, the `Fatrix` class), that provide a "fake matrix" object. These objects exploit MATLAB's object oriented features, specifically operator overloading. The following overview of these objects should help in understanding the reconstruction code.

3.1 The `strum` class

Most object-oriented languages allow object classes to have private methods, *i.e.*, functions that are intended for use only with objects or data of a specific class. MATLAB also supports the use of private methods. If you put a function in an m-file named `myfun.m` within the directory where an class `myclass` is declared, *i.e.*, in the directory `@myclass`, then invoking `myfun(ob)` will call the function `myfun` if `ob` is of class `myclass`. This mechanism is very convenient, particularly when overloading an existing MATLAB operation, but it has some limitations.

- If several object classes can all use the same method `myfun`, the you must put copies of `myfun` in each of the object class directories (or use suitable links), which complicates software maintenance.
- Every single method requires such an m-file, even if the function is only a few lines long, leading to a proliferation of little m-files littering the directories.
- There is no mechanism for changing the methods during execution.

The `strum` object class is essentially a special *structure* that contains private *methods* that are simply function handles.

If `st` is a `strum` object that was declared to have a method `method1`, then invoking

`st.method1(`*args*`)`

will cause a call to the function handle.

A concrete example is given in the `sino_geom.m`. A call of the form

```
sg = sino_geom('par', 'nb', 128, 'na', 100, 'dr', 3, 'orbit', 180);
```

creates a `strum` object that describes a parallel-beam sinogram. This object has a variety of data elements and associated methods. For example, invoking `sg.ar` returns a list of the projection view angles in radians, and `sg.ad(2)` returns the second projection view angle in degrees. These methods are very short functions defined within the `sino_geom.m` m-file.

3.2 The `fatrix2` class

Most iterative algorithms for image reconstruction are described conveniently using matrix notation, but matrices are not necessarily the most suitable data structure for actually implementing an iterative algorithm for large sized problems. The `fatrix2` class provides a convenient bridge between matrix notation and practical system models used for iterative image reconstruction.

Consider the simple iterative algorithm for reconstructing $\boldsymbol{x}$ from data $\boldsymbol{y}$ expressed mathematically:

$$\boldsymbol{x}^{n+1} = \boldsymbol{x}^n + \alpha \boldsymbol{A}'(\boldsymbol{y} - \boldsymbol{A}\boldsymbol{x}^n), \tag{1}$$

where $\boldsymbol{A}$ is the *system matrix* associated with the image reconstruction problem at hand. If $\boldsymbol{A}$ is small enough to be stored as a matrix in MATLAB (sparse or full), then this algorithm translates very nicely into MATLAB as follows.

```
x = x + alpha * A' * (y - A * x);        (2)
```

You really cannot get any closer connection between the math and the program than this! But often we work with system models that are too big to store as matrices (even sparsely) in MATLAB. Instead, the models are implemented by subroutines that compute the "forward projection" operation $\boldsymbol{A}\boldsymbol{x}$ and the "backprojection operation $\boldsymbol{A}'\boldsymbol{z}$ for input vectors $\boldsymbol{x}$ and $\boldsymbol{z}$ respectively. The conventional way to use one of these system models in MATLAB (or C) would be to rewrite the above program as follows.

```
Ax = forward_project(system_arguments, x)
residual = y - Ax;
correction = back_project(system_arguments, residual)
x = x + alpha * correction
```

Yuch! This is displeasing for two reasons. First, the code looks a *lot* less like the mathematics. Second, usually you end up with a different version of the code for every different system model (forward/back-projector subroutine pair) that you develop. Having multiple versions of a simple algorithm creates a software maintenance headache.

The elegant solution is to develop MATLAB objects that know how to perform the following operations:

- `A * x` (matrix vector multiplication, operation `mtimes`)
- `A'` (`transpose`), and
- `A' * z` (`mtimes` again, with a transposed object).

Once such an object is defined, one can use *exactly* the same iterative algorithm that one would have used with an ordinary matrix, *e.g.*, (2). The `fatrix2` class provides a convenient mechanism for implementing such linear operators. Specifically, suppose $\boldsymbol{x}$ is of length 1000 and $\boldsymbol{y}$ is of length 2000. Then use the following call:

```
A = fatrix2('imask', [1000 1], 'omask', [2000 1], ...
        'arg', system_arguments, ...
        'forw', @forward_project, 'back', @back_project);
```

The resulting `fatrix2` object `A` acts just like a matrix in most important respects. In particular, we can use exactly the same iterative algorithm (1) as before, because `A * x` is handled internally by calling

```
Ax = forward_project(system_arguments, x)
```

and similarly for `A' * z`.

Basic operations like `A(:,7)` are also implemented, but nonlinear operations like `exp(A)` are not because those cannot be computed readily using `forward_project`.

For many examples, see the `systems` subdirectory of IRT.

3.2.1 Operations on a **`fatrix2`** object that return another **`fatrix2`** object

- `B = A'` or `B = ctranspose{A}`
  `fatrix2` object Hermitian transpose
- `C = A * B`
  `fatrix2` object multiplication (requires compatible sizes)
- `B = 7 * A`
  multiplying a scalar times a `fatrix2` object
- `A = [A1; A2; A3]`
  vertical concatenation (`vertcat` is also supported) (requires compatible sizes)
- `A = [A1, A2, A3]`
  horizontal concatenation (`horzcat` is also supported) (requires compatible sizes)
- `A(:,[3 7])` or `A([3 7],:)` or `A(:,2:end)` etc.
  these return a smaller sized `fatrix2`
- `C = B + A`
  `fatrix2` object addition (requires compatible sizes)

3.2.2 Operations on a **`fatrix2`** object that return a vector.

- `A(:,7)` or `A(7,:)`

3.2.3 Operations on a **`fatrix2`** object that return a matrix.

These are practical only if `A` has sufficiently small size!

- `A(:,:)` or `full(A)`
- `svd(A)`
- `eig(A)`

3.2.4 Support for arrays instead of columns

For an image reconstruction problem with the mask illustrated in Fig. 1, the size of `A` should be $n_\mathrm{d} \times 8$ where n_d is the number of rows of $\boldsymbol{A}$.

So if we have a 6×5 image `x` and we would like to compute $\boldsymbol{A}\boldsymbol{x}$, conventionally one would need to do the following:

```
y = A * x(mask)
```

The statement `x(mask)` extracts the relevant $n_\mathrm{p} = 8$ values out of the 6×5 array `x`.

Objects in the `fatrix2` class often can spare the user the need to use `x(mask)` if the object is defined properly.

Suppose that in a tomographic image reconstruction problem corresponding to Fig. 1, the sinogram size is 9×8 so $n_\mathrm{d} = 72$.

Then if `A` is one of the predefined tomographic system models in IRT such as `Gtomo2_wtmex`, then the statement

```
yc = A * x(mask)
```

will produce *column vector* `yc` with $n_\mathrm{d} = 72$ elements.

On the other hand, the convenient syntax

```
ya = A * x
```

will produce a 9×8 sinogram *array* output `ya`. The two different outputs are related by

```
yc = ya(:)
```

In other words, if the input is a column vector (of length n_p), then the output will also be a column vector, just like one would expect for an ordinary matrix. But if the input is an *array*, of the appropriate dimensions, then the output will also be an array.

When defining a new `fatrix2` object, there are several options that one can use to specify the appropriate dimensions.

- `imask` is the usual input mask. Default is `true(idim)`
  This can also be called just `mask` for backwards compatibility with `Fatrix` objects.
- `idim` describes the input array dimensions. Default is `sum(imask(:))`, *i.e.*, an ordinary column vector.
- `omask` is a rarely used option. Default is `true(odim)`
- `odim` describes the output array dimensions. Default is `sum(omask(:))`, *i.e.*, an ordinary column vector.

For a typical `fatrix2` object, one will use only the `imask` and `odim` options.

For the tomography example above, we would add the name-value pairs `'odim', [9 8]` to the `fatrix2` call.

3.2.5 Defining `fatrix2` methods

The key methods for a `fatrix2` object are the `forw` and `back` operations. For the obsolete `Fatrix` objects, these methods had to support columns and arrays and multiples thereof which made them fairly complicated. For the new `fatrix2` objects, this complexity is handled by the object itself, and the user-defined methods are much simpler.

The `forw` routine accepts a single input array of size `idim` and returns a single output array of size `odim`.

(If there are nonzero values in the input array outside of `imask` then the output results may be unpredictable

Conversely, the `back` routine accepts a single input array of size `odim` and returns a single output array of size `idim`.

Caution: the output array of the `back` routine must be zero for any pixels outsize of the `omask`.

Unfortunately, MATLAB does not really support 1D arrays. (MATLAB's `size` command always returns at least a two-element vectors.) So for any application where `odim` is 1D, such as MRI with irregular non-Cartesian k-space samples, `A' * y` will produce a 1D array not a 2D array even if `imask` is 2D, because `y` will be 1D in such cases.

3.3 The `Fatrix` class

Most iterative algorithms for image reconstruction are described conveniently using matrix notation, but matrices are not necessarily the most suitable data structure for actually implementing an iterative algorithm for large sized problems. The `Fatrix` class provides a convenient bridge between matrix notation and practical system models used for iterative image reconstruction.

Consider the simple iterative algorithm for reconstructing $\boldsymbol{x}$ from data $\boldsymbol{y}$ expressed mathematically:

$$\boldsymbol{x}^{n+1} = \boldsymbol{x}^n + \alpha \boldsymbol{A}'(\boldsymbol{y} - \boldsymbol{A}\boldsymbol{x}^n), \tag{3}$$

where $\boldsymbol{A}$ is the *system matrix* associated with the image reconstruction problem at hand. If $\boldsymbol{A}$ is small enough to be stored as a matrix in MATLAB (sparse or full), then this algorithm translates very nicely into MATLAB as follows.

```
x = x + alpha * A' * (y - A * x);    (4)
```

You really cannot get any closer connection between the math and the program than this! But these days we often work with system models that are too big to store as matrices in MATLAB. Instead, the models are represented by subroutines that compute the "forward projection" operation $\boldsymbol{A}\boldsymbol{x}$ and the "backprojection operation $\boldsymbol{A}'\boldsymbol{z}$ for input vectors $\boldsymbol{x}$ and $\boldsymbol{z}$ respectively. The conventional way to use one of these systems in MATLAB (or C) would be to rewrite the above program as follows.

```
Ax = forward_project(x, system_arguments)
residual = y - Ax;
correction = back_project(residual, system_arguments)
x = x + alpha * correction
```

Yuch! This is displeasing for two reasons. First, the code looks a *lot* less like the mathematics. Second, you need a different version of the code for every different system model (forward/back-projector pair) that you develop. Having multiple versions of a simple algorithm creates a software maintenance headache.

The elegant solution is to develop MATLAB objects that know how to perform the following operations:

- `A * x` (matrix vector multiplication, operation `mtimes`)
- `A'` (`transpose`), and
- `A' * z` (`mtimes` again, with a transposed object).

Once such an object is defined, one can use *exactly* the same iterative algorithm that one would have used with an ordinary matrix, *e.g.*, (4). The `Fatrix` class provides a convenient mechanism for implementing such linear operators. Specifically, suppose $\boldsymbol{x}$ is of length 1000 and $\boldsymbol{y}$ is of length 2000. Then use the following call:

```
A = Fatrix([2000 1000], system_arguments, 'forw', @forward_project, 'back', @back_project);
```

The restulting `Fatrix` object `A` acts just like a matrix in most important respects. In particular, we can use exactly the same iterative algorithm (3) as before, because `A * x` is handled internally by calling

```
Ax = forward_project(x, system_arguments)
```

and similarly for `A' * z`.

Basic operations like `A(:,7)` are also implemented, but nonlinear operations like `A .^ 1/3` are not because those cannot be computed readily using `forward_project`.

For examples, see the `systems` subdirectory of IRT.

On 2007-1-30, inspired by `bbtools`, I added the following functionality:

- Fatrix object multiplication (using `Gcascade`): `C = A * B`
- Scalar multiplication of Fatrix object (using `Gcascade`): `B = 7 * A`
- Vertical concatenation (using `block_fatrix`): `A = [A1; A2; A3]`
- Horizontal concatenation (using `block_fatrix`): `A = [A1, A2, A3]`

One could use `Gcascade` or `block_fatrix` directly for these operations, but it looks nicer and is more "MATLAB like" to use the new syntax.

3.4 Related tools

On 2007-1-28, I noticed that there is another package called `bbtools` at `http://nru.dk/bbtools` that has a similar functionality called a "black box." It is nicely documented.

On 2010-02-22 I became aware of the "SPOT" package that has some similar functionality. `http://www.cs.ubc.ca/labs/scl/s`[illegible]
