# Supplementary material for: Multichannel Compressive Sensing MRI Using Noiselet Encoding
Source: PLoS One. 2015 May 12;10(5):e0126386. doi: 10.1371/journal.pone.0126386 (PMC4429034; doi:10.1371/journal.pone.0126386)
Supplement: S1 Matlab Code — (ZIP) [file pone.0126386.s001.zip › code/irt/mex/src/fdk/fdk-cuda-wei/doc/cuda-book-notes.docx]

# Chapter 3: Introduction to CUDA

- Host: CPU (e.g. personal computer)
- Devices: massively parallel processors with a large number of arithmetic execution units

## 3.1 Data Parallelism

- Images and video frames are snapshots of a physical world where different parts of a picture capture simultaneous, independent physical events
- Parallelism: many arithmetic operations can be safely performed on the data structures in a simultaneous manner.
- Example: dot product of matrices. A 1000x1000 matrix multiplication has 1,000,000 dot products, each involving 1000 multiply and 1000 accumulate arithmetic operations.

## 3.2 CUDA Program Structure

- A CUDA program consists of one or more phases
  - Phases that exhibit little or no data parallelism are implemented in host code
  - Phases that exhibit rich amount of data parallelism are implemented in device code
- A CUDA program is a unified source code encompassing both host and device code: NVIDIA C compiler (nvcc) separates the two during compilation.
  - Host code is ANSI C code, compiled with host’s standard C compiler, run as ordinary CPU process
  - Device code is written using ANSI C extended with keywords for labeling **data-parallel functions (called kernels)** and their associated data structures, compiled by nvcc and executed on GPU (or on CPU using emulation features such as SDK or MCUDA tool).
- Kernal function example: matrix multiplication
  - Entire calculation in a kernel, uses one thread to compute each output
  - Number of thread used in kernel is a function of matrix dimension. For 1000x1000 matrix multiplication, kernel would generate 1,000,000 threads
- Threads take very few cycles comparing to CPU threads
- **All the threads that are generated by a kernel during an invocation are collectively called a grid**
- CPU serial code → GPU parallel code (grid 0) kernalA<<<nBIK,nTID>>>(args); → CPU serial code → GPU parallel code (grid 1) (p. 42 figure 3.2)

## 3.3 A Matrix-Matrix Multiplication Example

int main (void) { (APPENDIX A for complete code)

1. Allocate and initialize the matrices M, N, P

I/O to read the input matrices M and N

2. M*N on the device

MatrixMultiplication(M,N,P,Width);

3. I/O to write the output matrix P

Free matrices M,N,P

Return 0; }

- MatrixMultiplication(): CPU only way requires 3 loops of matrix width elements
- Modify MatrixMultiplication() to be done on GPU:

void MatrixMultiplication( float* M, float* N, float* P, int Width){

int size = Width*Width*sizeof(float);

float *Md, Nd, Pd;

1. Allocate device memory for M,N,P

Copy M and N to allocated device memory locations

2. Kernel invocation code – to have the device to perform the actual matrix multiplication
3. Copy P from the device memory

Free device matrices	}

Need: Placement of 2-D array elements into the linear address system memory fig.3.5

- My addVec() example:

1. allocating CPU memory
2. allocating GPU memory
3. copying input data to GPU mem
4. parallel operation on GPU (n number of threads)
5. Reading back GPU result
6. Freeing GPU memory

## 3.4 Device Memories and Data Transfer

cudaMalloc() (must cast pointer variables to (void**), cudaMemcpy(), cudaFree()

FIGURES FROM THIS SECTION

## 3.5 Kernel Functions and Threading

- A kernel function specifies the code to be executed by all threads during a parallel phase
- All threads execute same code, CUDA is an instance of single-program multiple-data (SPMD) style
- _global_ : indicates that the function being declared is a CUDA kernel function, will be executed on device and can only be called from the host to generate a grid of threads on a device.
- By default, all functions in a CUDA program are _host_ functions if no keywords
- _host_: executed on the host, only callable from the host
- _device_: executed on the device, only callable from the device
- One can use both _host_ and _device_ keywords, compiles 2 versions of function
- threadIdx.x, threadIdx.y: thread indices of a thread
- blockIdx.x, blockIdx.y: each block is organized as a 3-D array of threads with a total size of up to 512 threads (x,y,z)
- If not defined, using only 1 block by default (can have up to 512 threads): NOT ACCEPTABLE

## 3.6 Summary

### 3.6.1 Function declarations

_global_, _device_, _host_

### 3.6.2 Kernel launch

<<< and >>> to define dimension of grid

### 3.6.3 Predefined variables

threadIdx, blockIdx, gridDim, blockDim

### 3.6.4 Runtime API

cudaMalloc(), cudaMemcpy(), etc

# Chapter 4: CUDA Threads

This chapter presents more details on the organization, resource assignment, and scheduling of threads in a grid. A CUDA programmer who understands these details is well equipped to write and to understand high-performance CUDA applications.

## 4.1 CUDA Thread Organization

- Because all threads in a grid execute the same code, they rely on unique coordinates to distinguish themselves from each other
- Thread ID = blockId .x * blockDim.x + threadIdx.x for example on FIGURE 4.1
- A grid has a 2D array blocks, each block is organized into a 3D array of threads
- Exact organization of grid is determined by the execution configuration provided at kernel launch.
- Example: FIGURE 4.1 organization and there are N=128 blocks, each block has M=32 threads
  - Host code to launch the kernel is:

dim3 dimGrid(128, 1, 1);

dim3 dimBlock(32,1,1);

kernelFunction<<<dimGrid, dimBlock>>>(…);

- Note: dim3 type is C struct with 3 unsigned integer fields: x, y, z for dimensions of blocks and threads
- Grids are 2D arrays of blocks, third field of grid dimension if always 1 (or ignored?)
- Values of gridDim.x and gridDim.y can range from 1 to 65,535 (16-bit integers)
- Total size of block is limited to 512 threads
- Multidimensional example of CUDA grid organization (FIGURE4.2)

## 4.2 Using blockIdx and theradIdx

- Matrix multiplication P=M*N example revisited: to allow larger matrices (>512 elements):
  - Break P into small tiles, each tile in a block (figure 4.3).
  - Use threadIdx and blockIdx to calculate the P element to work on

## 4.3 Synchronization and Transparent Scalability

- Barrier synchronization function, _syncthreads()
  - Thread execute function call held at calling location until every thread in block reaches location
- Barrier synchronization to organize parallel activities: for example, friends going to different stores at the mall but wait by the car when they are done
- In CUDA, a _syncthreads() statement must be executed by all threads in a block
  - If statement: either all threads in a block execute syncthreads() or none of them does
  - If-then-else statement: all threads in a block execute syncthreads() in “then” all execute syncthreads() in “else”, the two are DIFFERENT (if execute different ones, they will be waiting at different barrier synchronization points and waiting FOREVER)
  - Be VERY AFRAID when you code
- Not allowing threads in different blocks to perform barrier synchronization, CUDA can execute blocks in any order relative to each other without having to wait for each other
  - Depends on resources (cost, power) and performance requirement, one can execute different number of blocks at the same time
  - Transparent scalability: allow same application code to run on hardware with different numbers of execution resources (figure 4.8)

## 4.4 Thread Assignment

- Execution resources are organized into streaming multiprocessors (SMs)
  - WE DO NOT get to organize them
- For example, NVDIA GT200 implementation has 30 SMs, up to 8 blocks can be assigned to each SM, up to 1024 threads (1 to 8 blocks depending on how many threads are in each block) per SM
  - Up to 1024x30=30,720 threads can be simultaneously residing in the SMs for execution, for NVIDIA G80, only 768x16SMs=12,288 threads simultaneously residing in SMs, but same code can run on both because of transparent scalability!!
  - If insufficient resources, CUDA runtime automatically reduces the number of blocks assigned to each SM until resources usage under limit
  - If more than 240 blocks, runtime system assign new blocks to SMs as they complete execution
  - (figure 4.9 and SM pdf)

## 4.5 Thread Scheduling and Latency Tolerance

- Thread scheduling: strictly an implementation concept, in context of specific hardware implementations only
- In GT200, once a block is assigned to a streaming multiprocessor, it is further divided into 32-thread unit called warps (figure 4.10 for streaming multiprocessor)
- Warps are used for efficient execution of long-latency operations (such as global memory accesses)
  - Latency hiding: filling the latency of expensive operations with work from other threads. When an instruction executed by the threads in a warp is waiting for a previously initiated long-latency operation another warp that is not waiting for results is selected for execution.
- With enough warps around, hardware will likely find a warp to execute at any point in time, making full use of the execution hardware in spite of long-latency operations.
- As a result of ability to tolerate long-latency operations, GPUs do not dedicate as much chip area to cache memories and branch prediction mechanisms as GPUs.
- GPUs dedicate more chip area to floating-point execution resources
- EXERCISE: for matrix multiplication, should we use 8x8, 16x16 or 32x32 thread blocks for GT200?
  - 8x8 blocks: 64 threads each block, need 1024/64=12 blocks to occupy an SM but not possible, we will end up using only 64x8=512 threads in each SM. SM execution resources will likely be underutilized because there will be fewer warps to schedule around long-latency operations
  - 16x16 blocks: 256 threads each block, need 1024/256=4blocks which is within 8 blocks limitation. This is good.
  - 32x32 blocks: 1024 threads each block, not possible because limit of up to 512 threads per block

## 4.6 Summary

- Only safe way for threads in different blocks to synchronize with each other is to terminate the kernel and start a new kernel for the activities after the synchronization point

## 4.7 Exercises

# CUDA Memories

- The poor performance is due to the fact that global memory (DRAM) tends to have long access latencies (hundreds of clock cycles) and finite access bandwitdth.
- Although having many threads available for execution can theoretically tolerate long memory access latencies (latency hiding from 4.5), one can easily run into a situation where traffic congestion in global memory access path prevents all but a few threads from making progress, thus rendering some SMs idle.
- This chapter: learn to use additional methods for accessing memory that can remove the majority of data requests to the global memory

## 5.1 Importance of Memory Access Efficiency

- In matrix calculation example, the most important part of the kernel in terms of execution time is the for loop that performs dot product.
- Every iteration, 2 global memory accesses (1 element from M and 1 from N) are performed for one floating-point multiplication and one floating-point addition
  - Thus, ratio of floating-point calculation to the global memory access operation is 1 to 1, or 1.0
- This above ratio is computer to global memory access (CGMA) ratio: number of floating point calculation performed for each access to the global memory within a region of a CUDA program
- CGMA important because devices have limited global memory access bandwidth (86.4 GB/s for NVIDIA G80)
- Highest achievable floating-point calculation throughput is limited by the rate at which the input data can be loaded from the global memory
- Will need to increase CGMA ratio (have more calculations for each global memory access) to achieve a higher level of performance for the kernel

## 5.2 CUDA Device Memory Types

- See FIGURE 5.2 for CUDA device memories
- Constant memory supports short-latency, high-bandwidth, read-only access by the device when all threads simultaneously access the same location
- Application programming interface (API) functions
- Registers and shared memory are on-chip memories
  - Variables that reside in these types of memory can be accessed at very high speed in a highly parallel manner
- Registers are typically used to hold frequently accessed variables that are private to each thread
- Shared memory is an efficient means for threads to cooperate by sharing their input data and the intermediate results of their work.
- See TABLE 5.1 for how to declare, memory, scope and lifetime
- If a variable’s lifetime is within a kernel invocation, it must be declared within the kernel function body and will only be available for use only by the kernel’s code
  - If kernel invoked several times, contents of variable are not maintained across these invocation
- If a variable’s lifetime is throughout the entire application, it must be declared outside of any function body.
  - Contents of the variable are maintained throughout the execution of the application and are available to all kernels
- Often use shared memory to hold the portion of global memory data that are heavily used in an execution phase of the kernel
  - One may need to adjust the algorithms used to create execution phases that focus heavily on small portions of the global memory data
- Constant variables are often used for variables that provide input values to kernel functions
  - Cached for efficient access
- Global variables are often used to pass information from one kernel invocation to another kernel invocation
- Note: there is a limitation on the use of pointer with CUDA variables declared in device memory.
  - In general, pointers are used to point to data objects in global memory
  - Two typical ways
    - Object allocated by host function (M, N, P)
    - Float *Ptr = &globalVar; assign address of globalVar into automatic point variable Ptr

## 5.3 A Strategy for Reducing Global Memory Traffic

- A common strategy is to partition the data into subsets called tiles such that each tile fits into the shared memory.
- Only works if kernel computations on these tiles can be done independently of each other (ot always the case)

## 5.4 Memory as a Limiting Factor to Parallelism

## 5.5 Summary

## 5.6 Exercises
