# Supplementary material for: Multichannel Compressive Sensing MRI Using Noiselet Encoding
Source: PLoS One. 2015 May 12;10(5):e0126386. doi: 10.1371/journal.pone.0126386 (PMC4429034; doi:10.1371/journal.pone.0126386)
Supplement: S1 Matlab Code — (ZIP) [file pone.0126386.s001.zip › code/irt/mex/src/fdk/fdk-cuda-wei/doc/project-report.docx]

University of Michigan

Back-Projection on GPU: Improving the Performance

EECS 499 Independent Study

**Wenlay “Esther” Wei**

**4/29/2010**

The purpose of this project is to accelerate the processing speed of the back-projection portion of Feldkamp-Davis-Kreiss (FDK) cone-beam image reconstruction algorithm implemented on a Graphics Processing Unit (GPU). This report summarizes, explains and analyzes each strategy that was tried as well as the findings when working on this project.

Table of Contents

[Introduction	2](#_Toc260234047)

[Problem Description	2](#_Toc260234048)

[Progress towards a Solution	3](#_Toc260234049)

[Strategy 1: Parallelization along Z-Axis (Bare minimum)	3](#_Toc260234050)

[Strategy 2: Projection View Data in Shared Memory (stepping up my skillz)	5](#_Toc260234051)

[Strategy 3: Reconstructing Each Voxel in Parallel (reach for the starz)	7](#_Toc260234052)

[Strategy 4: Shared Memory Integration between Two Kernels (classified)	8](#_Toc260234053)

[Strategies Not Used	9](#_Toc260234054)

[Resolving Thread Divergence	9](#_Toc260234055)

[Constant Memory	10](#_Toc260234056)

[Constraints	10](#_Toc260234057)

[Conclusion	11](#_Toc260234058)

[References	11](#_Toc260234059)

[Appendix	11](#_Toc260234060)

# Introduction

Feldkamp-Davis-Kreiss (FDK) algorithm is a filtered back-projection algorithm for 3D image reconstruction from 2D cone-beam projections. This algorithm is used for Computed Tomography (CT) reconstruction. This algorithm is to be implemented on a Graphic Processing Unit (GPU) which features parallel computing. Theoretically, using a GPU is ideal for image processing since parallelism can potentially decrease the processing time as parts of the image can be reconstructed simultaneously. In order to take advantage of parallelism, one needs to understand the architecture of GPU and CUDA language in order to write the most efficient program. A few students have developed a set of working CUDA code for FDK algorithm. My goal in this project is to improve the performance and accelerate the processing speed of this set of code.

# Problem Description

The original CUDA code implemented FDK algorithm by invoking a kernel that contains the dimension of the object (i.e. image) along x-axis (the value is called nx) times image’s dimension along y-axis (ny) threads total. The threads are organized in 16 by 16 blocks of threads. Each thread is reconstructs one “bar” of voxels with the same (x,y) coordinates of the 3D image. It loops through the dimension of the image along image’s z-axis (nz) to reconstruct each voxel therefore each thread reconstructs nz voxels (Figure 1). The kernel is executed for each view and the back-projection result is added onto the image.

When reconstructing a 128x124x120-voxel image from a 224x220-pixel projection view using this code, we were able reduce the processing time by 2.2 fold (1.570 seconds versus 3.369 seconds) compared to the implementation that solely runs on the CPU. The purpose of my project is to accelerate the processing speed even further.

Figure 1: Kernel diagram of original code

# Progress towards a Solution

Below are the summaries and analyses of each strategy I used to improve the original given CUDA code and why each method succeeded or failed. At the end of this section I will explain the strategies that I considered but ended up not using for this project and the reasons I did not choose those strategies.

## Strategy 1: Parallelization along Z-Axis

The motivation behind this strategy is the fact that even though the original CUDA code implemented the back-projection of each (x,y) pair of the image in parallel, it contains a loop that iterates through all the z values. The iteration through z values reconstructs nz voxels sequentially. The reason behind this design is the fact that since every voxel with the same (x,y) index of image coordinates corresponds to the same s index of the detector coordinates on the projection view, reconstructing each voxel in each thread would result in a lot of repeating computations (nz repetitions of operations that calculate s indices). However, these voxels correspond to different t indices of the detector coordinates (requires a different portion of the projection view along the t-axis) and the reconstruction of each of these voxels is independent of each other even though they share the same (x,y) index; this provides an opportunity to parallelize the process further.

In order to parallelize the reconstruction of each voxel along the z-axis and avoid repeating the computations of the same s index, an additional kernel is needed. The first kernel contains nx*ny threads and performs the calculations that are shared between voxels with the same (x,y) index. The second kernel contains nx*ny*nz threads and reconstructs each voxel of the image (Figure 2). The parameters that are shared among all voxels with the same (x,y) index (there are three shared parameters: ss_bin, w2, mag) are stored in the global memory at the end of the first kernel execution and loaded to the local registers at the beginning of the second kernel execution.

Figure 2: Diagram of kernels for Strategy 1

Reconstruction of a 128x124x120-voxel image using this method results in a processing time reduction of 2.5 fold compared to the pure CPU version (1.358 seconds versus 3.888 seconds). This is an improvement compared to the original method. Running the CUDA Profiler to analyze the results allows us to see that the GPU spent most of the time in kernel 2 (Figure 3) and that the global memory access time dominates the kernel 2 process (Figure 4). The details on how to use the profiler is recorded in Appendix A. Even though the sequential process from the original code is eliminated using this strategy, the global memory accesses that are required to integrate the two kernels prevents this method from an even greater speed-up.

Note: It is commonly assumed that as the dimensions of the image increase, the time spent on instructions will dominate the global memory accesses, allowing us to obtain a greater speed-up compared to the pure CPU version. However, the reconstruction of a 256x248x240-voxel image using this method did not result in a higher speed up. Please refer to Appendix B for more details.

Figure 3: GPU Time Summary of Strategy 1

Figure 4: GPU time breakdown of Strategy 1 kernel 2

## Strategy 2: Projection View Data in Shared Memory

Strategy 1 (parallelizing along the z-axis) improved the performance of the original code. However, the global memory loads and stores occupy the second kernel 28% of the processing time. In order to reduce the frequency of costly global memory accesses, shared memory can be used to store data shared between threads in the same block (Figure 5). This strategy is a modified version of previous strategy.

Figure 5: CUDA memory model

The idea of this strategy is to copy the projection view data that is stored in the global memory into shared memory since the data is being loaded four times in a given thread of kernel 2 (Strategy 1 code). The threads that share the same projection view data are grouped in the same block and every thread is responsible for copying a portion of the projection view data from the global memory to shared memory. As long as the number of global memory loads is less than four, this method will be considered an improvement over the previous.

It turns out that since the reconstruction algorithm takes in four neighboring pixels from the projection view on the detector and weighs them, it is rather complicated to predict which section of data is needed for each block. For the example of 128x124x120-voxel image and 224x220-pixel projection view that I have been using, there are 220*2=440 pixels needed for one block (fixed (x,y) values and variable z since they share the same s index) in order to take the neighboring pixels into consideration. There are only 120 voxels along the z axis and nz does not equal to nt (number of pixels along t-axis on the detector). As result, it is necessary for each thread to copy four pixels from the global memory into shared memory otherwise the results would be approximate. It would cost the same processing time for memory accesses as the previous strategy to copy from the global memory four times and, on top of that, there are complex algorithms involved to determine the projection view data to copy. I chose the second method and allowed each thread to copy only two pixels from the global memory into shared memory. My attempt resulted in an algorithm that does not reconstruct the image correctly (code is included in the folder). It can be concluded that in order to implement the algorithm correctly using shared memory for projection data, it would cost the same in terms of global memory accesses as Strategy 1. In addition, there are complex algorithms involved in order to figure out which piece of projection view data to copy and which data in the shared memory to use.

## Strategy 3: Reconstructing Each Voxel in Parallel

Global memory loads and stores are costly operations in terms of processing time for Strategy 1 and Strategy 2 did not improve the performance of the Strategy 1 algorithm. Even though algorithm 1 eliminates the sequential process of the original code, it introduces a global memory store of three times per thread in kernel 1 and a global memory load of three times per thread in kernel 2. Strategy 3 trades the processing time spent on these global memory accesses with the processing time spent on repeated instructions. This method performs reconstruction on each voxel in parallel completely (Figure 6). Kernel 1 and kernel 2 from Strategy 1 are combined into one kernel of nx*ny*nz threads and the repeated computation of voxels that share the same (x,y) image coordinates is introduced.

Figure 6: Kernel diagram of Strategy 3

The eliminated global memory access time does compensate for the processing time of repeated computation. However, it does not improve the performance overall. The speed-up of reconstructing a 128x124x120-voxel image with this strategy compared to the CPU version is 2.5 times (1.507 seconds versus 3.782 seconds), which is similar to the speed-up for Strategy 1. Figure 7 shows that the kernel spends more time on instructions (repeating computations) than on global memory accesses.

Figure 7: GPU Time breakdown of Strategy 3 kernel

## Strategy 4: Shared Memory Integration between Two Kernels

Reconstructing each voxel in parallel (Strategy 3) does not result in better performance than that of Strategy 1. Therefore, it is more appropriate to modify Strategy 1 in order to reduce the time spent on global memory accesses.

In order to avoid sequential processes (as in the original code) as well as repeated computations (as in Strategy 3), it is necessary to have two kernels. Since at least three parameters are shared between two kernels, global memory accesses cannot be avoided. This strategy follows the same kernel organization as Strategy 1 (Figure 2), but the integration between two kernels is modified as shared memory is used. The first kernel stores these data into global memory and, instead of having each thread of the second kernel load the data from global memory individually (as in Strategy 1), the threads are arranged in a way that all the threads sharing the same data from global memory reside in the same block so that only the first thread has to load the data from global memory into shared memory. Since all these threads use the same data and shared memory is accessible for every thread within the same block, the frequency of global memory loads is reduced greatly. Since the parameters (ss_bin, w2, mag) passed on between the two kernels are the same for reconstruction of all the voxels with the same (x,y) image index, the threads for these voxels are grouped in the same block. As a side note, it is important to synchronize all the threads within a block after loading data from global memory into shared memory so that no threads will attempt to access the shared memory before the global memory loads, resulting in loading incorrect data.

Reconstruction of a 128x124x120-voxel image using this method results in a processing time reduction of 7 fold compared to the pure CPU version (0.502 seconds versus 3.513 seconds). This is a significant improvement compared to the 2.2-fold speed-up of the original method. Reconstructing a larger image of 256x248x240 voxels with this method results in 8.5 times of processing time reduction (6.547 seconds versus 55.327 seconds). The same method is used to reconstruct a 512x496x480-voxel image and a 7.9-fold speed-up compared to the pure CPU version (107.0 seconds versus 849.4 seconds) is obtained.

Running the CUDA Profiler to analyze the 128x124x120-voxel image reconstruction results allows us to see that GPU spent most of the time in kernel 2 (Figure 8), as expected, and that the instruction execution time occupies 44.5% of kernel 2 processing time while global memory loads occupy only 20% and global memory stores occupy even less than 10% (Figure 9). It is ideal that instruction execution time dominates memory access time because this means the GPU is spending most of the time doing useful work instead of loading and storing from memory.

Figure 8: GPU time summary for Strategy 4

Figure 9: GPU Time breakdown of Strategy 4 kernel 2

## Strategies Not Used

### Resolving Thread Divergence

GPU hardware executes threads with single-instruction, multiple thread (SIMT) style. That is, threads are organized into 32-thread warps. All the threads within the same warp execute the same instruction at a given time. In the case of branches, if all the threads within the same warp either take the branch or do not take the branch, then they will execute the same instruction as normal. However, if the branch is taken by some of the threads in a warp and not taken by the others, the branch-taken threads will execute instructions while the other threads wait and then the branch-not-taken threads will execute instructions. The diverging threads will execute each set of instructions in a sequential manner thus taking two passes for each divergence within a warp. There are several if statements in the original code checking if the thread is within the boundaries . I thought thread divergence would be a problem and was seeking solutions. However, upon the discovery of the CUDA Profiler, I learned that divergent branches only occupied less than 1% of GPU processing time of the dominant kernel. One of the reasons could be that most of the threads are well within the boundaries, therefore they follow the same path when branching. As a result, there are not many divergent threads in this project.

### Constant Memory

Constant memory is read-only memory that all threads in the same grid can load data from (Figure 5). It is accessible by both host (CPU) and device (GPU) and it allows for faster access than global memory. I considered copying all the projection view data into constant memory instead of global memory since it will not be modified by the GPU process. However, there are only 64 kilobytes (2_­_­­^16^ bytes) of constant memory in the GeForce GTX 260 GPU we are using (Figure 10). Each float is 4 bytes so the constant memory can contain 2^14^ floats. A 128x128 projection view uses that much memory and it is possible for the dimensions of projection view to be much larger than 128x128 pixels, therefore requiring larger memory. I concluded that constant memory is meant for constant variables and not large arrays.

Figure 10: GeForce GTX 260 properties

# Constraints

We are limited by the GPU properties (Figure 10). When considering a strategy to improve the performance of CUDA programs, we must also consider the feasibility of this strategy given the GPU. For example, constant memory is limited; therefore, we cannot store the projection view data in it. When designing Strategy 4, I decided to copy all the parameters common between kernel 1 and kernel 2 into shared memory. I did some calculations to make sure this is possible: there are 16 kilobytes (2^14^ bytes) of shared memory per each block and the memory needed for parameters data is only 3*4=12 bytes (each float takes up 4 bytes) for each block. This is definitely a feasible plan.

Another constraint that we must keep in mind is the maximum threads per block and the maximum block and grid dimensions (Figure 10). For example, I would like to keep all the threads that reconstruct the voxels with the same (x,y) coordinates in the same block—this would result in nz threads per block and an nx by ny grid. The maximum grid dimensions are 2_­_­­^16^x2_­_­­^16^, so the grid dimensions are definitely feasible. I noticed that the maximum z-dimension of a block is only 64; therefore, I stored the nz threads along the x-dimension, which allows 512 blocks. Also, there is a restriction on having a maximum of 512 threads per block. Therefore, nz must be less than or equal to 512 or additional logic is necessary to tile the blocks on the grid.

# Conclusion

In order to optimize the performance of a CUDA program, we must eliminate as many sequential processes as possible and avoid repeating multiple computations. This reduces processing time spent on huge numbers of instructions. Since global memory access is a time-costly process, the number of accesses should be kept to the minimum necessary to correctly perform the algorithm. One of the solutions is to use shared memory. We must carefully strategize the usage of shared memory in order to actually improve the performance while obtaining correct results. Minimizing global memory accesses will reduce processing time significantly in most cases.

When designing strategies to improve the performance, we should always consider if the strategy would work on the specific example we are working on. Just because a strategy improved the performance of a certain project does not mean that it will work well with another. We should always gather information on the performance of our specific program (perhaps by using the CUDA Profiler) in order to know which area has room for improvement. Lastly, we should always consider the physical constraints when designing CUDA programs in order to ensure that the design is feasible.

# References

Kirk, David, and Wen-mei Hwu. *Programming Massively Parallel Processors: a Hands-on Approach*. Burlington, MA: Morgan Kaufmann, 2010. Print.

Fessler, J. "Analytical Tomographic Image Reconstruction Methods." Print.

# Appendix

## A. CUDA Profiler

CUDA Profiler is located at /usr/local/cuda/cudaprof/bin

At the terminal, type in:

cd /usr/local/cuda/cudaprof/

./cudaprof

The CUDA Profiler window will pop up. Then start a New Session. Change the Session Settings to resemble the settings of the snapshot below:

“init.m” contains the following code:

function [ ] = init()

addpath('/net/escanaba/z/ewwei/testCode/alg');

addpath('/net/escanaba/z/ewwei/testCode/alg/mex/src/fdk-cuda');

setup;

cbct_back test;

quit;

Click “Start” and see results!

## B. MATLAB output when running cbct_back test

### Original Code

Downsampling rate = 4

```
cbct_back_test: [ig.nx ig.ny ig.nz ig.dx ig.dy ig.dz] =
  128.0000  124.0000  120.0000    3.9062   -3.9062    3.9062

cbct_back_test: [cg.ns cg.nt cg.na cg.ds cg.dt] =
  224.0000  200.0000  246.0000    4.0956    4.3856

cbct_back_test: image Mbytes 7.265625e+00
cbct_back_test: proj Mbytes 4.204102e+01
cbct_back_test: found cuda version
cbct_back_test: testing version:
	/net/escanaba/z/ewwei/testCode/alg/mex/v7/jf_mex.mexa64
cbct_back_test: time jf_mex = 3.36899
cbct_back_test: testing version:
	/net/escanaba/z/ewwei/testCode/alg/mex/src/fdk-cuda/fdk_mex.mexa64
cbct_back_test: time fdk_mex = 1.57033
max_percent_diff(, ) = 0.00256535%
```

### Strategy 1

Downsampling rate = 4

```
cbct_back test
cbct_back_test: [ig.nx ig.ny ig.nz ig.dx ig.dy ig.dz] =
  128.0000  124.0000  120.0000    3.9062   -3.9062    3.9062

cbct_back_test: [cg.ns cg.nt cg.na cg.ds cg.dt] =
  224.0000  200.0000  246.0000    4.0956    4.3856

cbct_back_test: image Mbytes 7.265625e+00
cbct_back_test: proj Mbytes 4.204102e+01
cbct_back_test: found cuda version
cbct_back_test: testing version:
	/net/escanaba/z/ewwei/testCode/alg/mex/v7/jf_mex.mexa64
cbct_back_test: time jf_mex = 3.38791
cbct_back_test: testing version:
	/net/escanaba/z/ewwei/testCode/alg/mex/src/fdk-cuda/fdk_mex.mexa64
cbct_back_test: time fdk_mex = 1.35808
max_percent_diff(, ) = 0.00256535%
```

Downsampling rate = 2

```
cbct_back_test: [ig.nx ig.ny ig.nz ig.dx ig.dy ig.dz] =
  256.0000  248.0000  240.0000    1.9531   -1.9531    1.9531

cbct_back_test: [cg.ns cg.nt cg.na cg.ds cg.dt] =
  444.0000  400.0000  492.0000    2.0478    2.1928

cbct_back_test: image Mbytes 5.812500e+01
cbct_back_test: proj Mbytes 3.333252e+02
cbct_back_test: found cuda version
cbct_back_test: testing version:
	/net/escanaba/z/ewwei/testCode/alg/mex/v7/jf_mex.mexa64
cbct_back_test: time jf_mex = 55.9977
cbct_back_test: testing version:
	/net/escanaba/z/ewwei/testCode/alg/mex/src/fdk-cuda/fdk_mex.mexa64
cbct_back_test: time fdk_mex = 25.3913
max_percent_diff(, ) = 0.00321063%
```

### **Strategy 3**

Downsampling rate = 4

```
cbct_back_test: [ig.nx ig.ny ig.nz ig.dx ig.dy ig.dz] =
  128.0000  124.0000  120.0000    3.9062   -3.9062    3.9062

cbct_back_test: [cg.ns cg.nt cg.na cg.ds cg.dt] =
  224.0000  200.0000  246.0000    4.0956    4.3856

cbct_back_test: image Mbytes 7.265625e+00
cbct_back_test: proj Mbytes 4.204102e+01
cbct_back_test: found cuda version
cbct_back_test: testing version:
	/net/escanaba/z/ewwei/testCode/alg/mex/v7/jf_mex.mexa64
cbct_back_test: time jf_mex = 3.78243
cbct_back_test: testing version:
	/net/escanaba/z/ewwei/testCode/alg/mex/src/fdk-cuda/fdk_mex.mexa64
cbct_back_test: time fdk_mex = 1.50728
max_percent_diff(, ) = 0.00256535%
```

### Strategy 4

Downsampling rate = 4

```
cbct_back test
cbct_back_test: [ig.nx ig.ny ig.nz ig.dx ig.dy ig.dz] =
  128.0000  124.0000  120.0000    3.9062   -3.9062    3.9062

cbct_back_test: [cg.ns cg.nt cg.na cg.ds cg.dt] =
  224.0000  200.0000  246.0000    4.0956    4.3856

cbct_back_test: image Mbytes 7.265625e+00
cbct_back_test: proj Mbytes 4.204102e+01
cbct_back_test: found cuda version
cbct_back_test: testing version:
	/net/escanaba/z/ewwei/testCode/alg/mex/v7/jf_mex.mexa64
cbct_back_test: time jf_mex = 3.51294
cbct_back_test: testing version:
	/net/escanaba/z/ewwei/testCode/alg/mex/src/fdk-cuda/fdk_mex.mexa64
cbct_back_test: time fdk_mex = 0.501608
max_percent_diff(, ) = 0.00256535%
```

Downsampling rate = 2

```
cbct_back_test: [ig.nx ig.ny ig.nz ig.dx ig.dy ig.dz] =
  256.0000  248.0000  240.0000    1.9531   -1.9531    1.9531

cbct_back_test: [cg.ns cg.nt cg.na cg.ds cg.dt] =
  444.0000  400.0000  492.0000    2.0478    2.1928

cbct_back_test: image Mbytes 5.812500e+01
cbct_back_test: proj Mbytes 3.333252e+02
cbct_back_test: found cuda version
cbct_back_test: testing version:
	/net/escanaba/z/ewwei/testCode/alg/mex/v7/jf_mex.mexa64
cbct_back_test: time jf_mex = 55.3267
cbct_back_test: testing version:
	/net/escanaba/z/ewwei/testCode/alg/mex/src/fdk-cuda/fdk_mex.mexa64
cbct_back_test: time fdk_mex = 6.54674
max_percent_diff(, ) = 0.00321063%
```

Downsampling rate = 1

```
cbct_back_test: [ig.nx ig.ny ig.nz ig.dx ig.dy ig.dz] =
  512.0000  496.0000  480.0000    0.9766   -0.9766    0.9766

cbct_back_test: [cg.ns cg.nt cg.na cg.ds cg.dt] =
  888.0000  800.0000  984.0000    1.0239    1.0964

cbct_back_test: image Mbytes 465
cbct_back_test: proj Mbytes 2.666602e+03
cbct_back_test: found cuda version
cbct_back_test: testing version:
	/net/escanaba/z/ewwei/testCode/alg/mex/v7/jf_mex.mexa64
cbct_back_test: time jf_mex = 849.422
cbct_back_test: testing version:
	/net/escanaba/z/ewwei/testCode/alg/mex/src/fdk-cuda/fdk_mex.mexa64
cbct_back_test: time fdk_mex = 107
max_percent_diff(, ) = 0.0114752%
```
